# Oxidative Stress in the Protection and Injury of the Lacrimal Gland and the Ocular Surface: are There Perspectives for Therapeutics?

**DOI:** 10.3389/fcell.2022.824726

**Published:** 2022-03-11

**Authors:** Camila Nunes Lemos, Lilian Eslaine Costa Mendes da Silva, Jacqueline Ferreira Faustino, Marina Zilio Fantucci, Adriana de Andrade Batista Murashima, Leidiane Adriano, Monica Alves, Eduardo Melani Rocha

**Affiliations:** ^1^ Department of Ophthalmology, Otorhinolaryngology and Head and Neck Surgery, Ribeirao Preto Medical School, University of Sao Paulo, Ribeirao Preto, Brazil; ^2^ Department of Ophthalmology and Otorhinolaryngology, Faculty of Medical Sciences, State University of Campinas (Unicamp), Campinas, Brazil

**Keywords:** lacrimal gland, ocular surface, oxidative stres, dry eye, lacrimal functional unit (LFU)

## Abstract

Oxidative stress (OS) is a major disruption in the physiology of the lacrimal functional unit (LFU). Antioxidant enzymes have dual protective activities: antioxidant and antimicrobial activities. Peroxidases have been indistinctly used as markers of the secretory activity of the LFU and implicated in the pathophysiology, diagnosis and treatment of dry eye disease (DED), even though they comprise a large family of enzymes that includes lactoperoxidase (LPO) and glutathione peroxidase (GPO), among others. Assays to measure and correlate OS with other local LFU phenomena have methodological limitations. Studies implicate molecules and reactions involved in OS as markers of homeostasis, and other studies identify them as part of the physiopathology of diseases. Despite these conflicting concepts and observations, it is clear that OS is influential in the development of DED. Moreover, many antioxidant strategies have been proposed for its treatment, including calorie restriction to nutritional supplementation. This review offers a critical analysis of the biological mechanisms, diagnostic outcomes, drug use, dietary supplements, and life habits that implicate the influence of OS on DED.

## Introduction

Oxidative stress (OS) takes part in both the protection and injury to the lacrimal functional unit (LFU) (Bron and Seal 1986; Stern et al., 2004; Davies et al., 2008; Uchino et al., 2012; Wakamatsu et al., 2013). It involves free radicals, which are atoms or molecules with a single unpaired electron composed of atoms of oxygen that react with other molecules donating or taking electrons and usually giving a considerable degree of reactivity to those molecules. The actions of these free radicals, named reactive oxygen species (ROS), are variable and influenced by several biological conditions (Petrides and Nauseef 2000; Droge 2002; Halliwell 2006).

At physiological concentrations, the ROS induces the appropriate level of OS required for a range of cellular processes, such as apoptosis, inflammation, innate immunity, and wound healing, as well as the regulation of transcription factors and their surface receptors (Halliwell 2006). However, excess ROS causes OS to oxidize DNA, protein, and other intracellular molecules. It also induces functional impairment, damage, or even the uncontrolled death of cells exposed to ROS, contributing to the mechanisms of several diseases, including eye diseases (Sies 1991; Halliwell and Chirico 1993; Halliwell 1995; Halliwell 2006; Ung et al., 2017).

The LFU is defined as a system that integrates the ocular surface, the central nervous system (CNS) and the exocrine glands responsible for the tear film. The ocular surface is composed of the cornea, the conjunctiva, and the lids that send sensorial neural inputs to the CNS, and throughout the autonomic nervous system, they provide feedback with stimuli to the exocrine glands, modulating the volume and content of the tear film that wets and nourishes the ocular surface. Among these exocrine glands are the lacrimal gland (LG), Meibomian glands (MG), accessory glands and the epithelial layer of the ocular surface, with their constitutive and regulated secretory and paracrine capacity (Bron and Seal 1986; Halliwell and Gutteridge 1990; Crouch et al., 1991; Droge 2002; Stern et al., 2004; Rocha et al., 2008).

OS is ubiquitous and has been implicated in the protective and pathogenic mechanisms of dry eye (DE) and ocular surface diseases. Therefore, any proposal of therapeutic modulation of those reactions must consider its dual effect (Morrison and Allen 1966; Fridovich 2013; Forman 2016).

As a protector, OS works against microorganisms and other sources of free radicals, maintaining homeostasis and triggering the response to protect against other harmful events that challenge the LFU. In a recent study on tear proteomics, the comparison between healthy and DE individuals revealed that lactoperoxidase (LPO) is the most downregulated enzyme in the DE group (Soria et al., 2017). In parallel, the increase in various proinflammatory proteins in their tears was confirmed in previous studies that used LPO as a biomarker of dysfunction for DE and LFU, as detailed below (Bromberg et al., 1989; Soria et al., 2017).

Throughout evolution, heme peroxidases, similar to myeloperoxidase (MPO), LPO, salivary peroxidase (SPO), and thyroid peroxidase (TPO), were found to be key enzymes for modulating the OS process in the cytoplasmic and secretory fluids (Davies et al., 2008). Antioxidants or free radical scavengers are incorporated into the innate immune arsenal, hormone synthesis and exocrine secretions, generating ROS with antimicrobial actions and counterbalancing the actions of other OS-related enzymes, including the glutathione peroxidases (GPx) family (Morrison and Allen 1966; Petrides and Nauseef 2000; Ihalin et al., 2006; Davies et al., 2008). Recently, a family of six protein isoforms called peroxiredoxins was described, and their structure and function associated with OS were further detailed (Rhee et al., 2012).

As a disease mediator, GPx levels and ROS accumulation have been identified as biomarkers and implicated in the protective mechanisms and damage triggered by the LFU (Wakamatsu et al., 2008). The expression and activity of these markers were associated with DE caused by hormone deficits, systemic autoimmune diseases, microelement deficiency, pollution, and ocular surface disease not necessarily related to systemic conditions (Bakker et al., 2005; Davies et al., 2008; Wakamatsu et al., 2008; Kawashima et al., 2010; Kojima et al., 2012; Uchino et al., 2012; Cejkova and Cejka 2015; Jung et al., 2018).

The present review aims to critically evaluate the apparent paradoxical protective and harmful aspects of ROS and the homeostatic or potentially therapeutic antioxidants associated with the ocular surface as well as LG protection and disease. Moreover, changes in the LFU related to OS, the reliability of the biomarkers, and the evidence supporting antioxidant therapies will be discussed herein.

## The Chemistry of Endogenous and Exogenous Reactive Oxygen Species Formation

As previously observed, free radicals composed of oxygen are mainly formed by an electron transfer reaction through several pathways, with the main group of ROS being formed during natural biological processes in aerobic cells. The eight main ROS formation pathways are 1) the mitochondrial electron transport chain; 2) cytochrome P-450 metabolism; 3) the autoxidation of catecholamines; 4) the oxidation of reduced nicotinamide adenine dinucleotide phosphate (NADPH) by NADPH oxidase; 5) the oxidation of xanthine or hypoxanthine by xanthine oxidase; 6) the reduction of molecular oxygen by NO synthase (NOS) isoforms (nNOS or eNOS) under arginine or tetrahydrobiopterin deficiency; 7) peroxisome metabolism and 8) the oxidation of transition heavy metals (Cu+ and Fe2+) (Kruk et al., 2015; Gebicki 2016; Ung et al., 2017; Kruk et al., 2019).

The exogenous sources of ROS include physical factors (sunlight, ionizing radiation, and gamma irradiation), persistent exposure to environmental chemicals (cigarette smoke, xenobiotics, aromatic amines, ozone, and organochlorines), and pathogens (Sies 1991; Sies 2015).

The main exogenous source of ROS formation in the eye is UV radiation. One of the possible causes of ROS formation is by chromophore molecule activation, such as flavins, porphyrin, tryptophan, and melanin, during photochemical reaction processes. Chemically, these reactions involve the electronic excitation of a chromophore to the singlet state, with subsequent conversion to the long-term triplet state. Then, the molecule interacts with an O_2_ molecule to form 1O_2_, returning afterward to its ground state. UV exposure can also generate • 2° through a common electron transfer reaction (Kruk et al., 2015).

## Historical Perspectives

The biological associations and medical implications of free radicals have drawn the attention of researchers since the 1950’s. They have been associated with the LG and LFU physiology and many diseases, including DE (Forman 2016; Dogru et al., 2018). Several enzymes linked to the redox process were identified in the LG and in tears, including the family of enzymes called catalases, superoxide dismutases and peroxidases.

**FIGURE 1:**
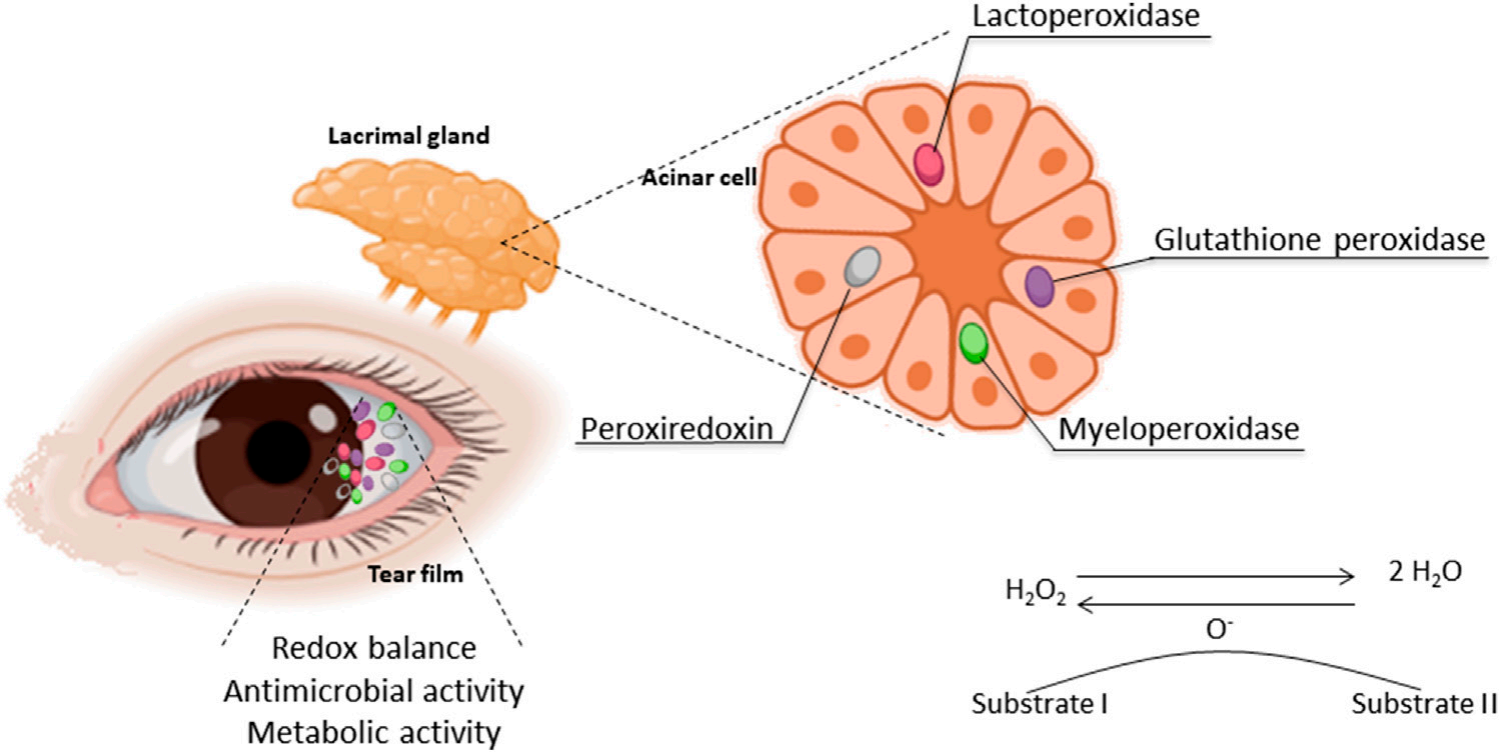
Oxidative stress in the LFU: the sources and area where the antioxidant enzymes take place and their activity.

Peroxidase is a general name for a large enzyme family that has been well preserved in the evolutionary process and is responsible for transforming hydrogen peroxide (H_2_O_2_) into water. By doing so, the levels of ROS are reduced in the cells. Data on peroxidase expression or activity in tears or in the LG as a marker of bioactivity of this exocrine gland have been published for more than 4 decades (Morrison and Allen 1966; Crouch et al., 1991) (Figure 1). It was immunologically detected in the LG of cows, and its expression and response to parasympathetic-like stimulation have also been observed in rats (Morrison and Allen 1966; Herzog and Miller 1972a; Herzog and Miller 1972b; Herzog et al., 1976).

Previous studies identified peroxidase granules in acinar cells to document the viability and maintenance of the secretory activity of those cells in culture conditions (Oliver et al., 1987). Aging has also been observed as a factor in the lowering of LG peroxidase secretion and activity in rats and mice (Bromberg and Welch 1985; Alves et al., 2005b; Rios et al., 2005). These observations made peroxidase activity a widely applied marker of LG bioactivity, not only in aging studies but also in other models investigating DE mechanisms, as detailed in the next section.

Earlier studies provided the opportunity to identify a marker of the structure and function of acinar cells and trace the secretory activity of those exocrine cells, measuring peroxidase in the tear film (Draper et al., 1999; Draper et al., 2003). However, the interpretation of these findings can be questioned. The studies assumed to measure secretory activity, and therefore the enzyme LPO, based on enzymatic assays of peroxidase activity in the tissues or secretion.

LPO is an antioxidant enzyme and member of the heme peroxidase superfamily II or the MPO family. This enzyme is constitutively secreted, which means it is present as a default component and is not determined by external demand or regulated processes. Its secretion occurs not only in tears but also in saliva, milk, and vaginal fluid. It is therefore considered to have a similar origin and structure as salivary peroxidase (SPO) (De et al., 1987; Ihalin et al., 2006). It oxidizes thiol from thiyl free radicals and participates in the host’s defense against bacteria.

Other enzymes associated with antimicrobial, antioxidant, and chemical scavenger functions were found in the LG, tear secretion, and the ocular surface. These include peroxidase isoforms, lactoferrin, and lysozyme, and their expression has been associated with age-related diseases (McGill et al., 1984; De et al., 1987; Gogia et al., 1998; Higuchi et al., 2012; Soria et al., 2017).

Studies performed in 1972 and 1991 failed to identify catalase in the tear film (Herzog and Miller 1972b; Crouch et al., 1991). However, enzymatic investigations using samples of the LG showed that those samples were capable of catalyzing the free radical hydrogen peroxide (H_2_O_2_) into water and oxygen *in vitro* after blocking peroxidase activity. This confirms the presence of catalase in the LG, assuming that it is a constitutive enzyme of this gland but not a substantial part of the secretory elements of the tear film (Herzog and Fahimi 1976). Recently, the presence of catalase was confirmed in the tear film by sensitive immunoassay analysis (Jee et al., 2014). Thus, it is possible to state that catalase is present in the LG, located in the peroxisomes in the cytoplasm, and that it is present in the tear film.

The presence of other soluble peroxidases in the LG and the ocular surface, including GPX, were noted to be abundant and have been described since 1996 (De 1992; Mazumdar et al., 1996).

Superoxide dismutase (SOD) is another family of enzymes capable of O_2_^−^ dismutation. It is a soluble protein present in the cytosol and nuclei of probably all mammalian cells (McCord and Fridovich 1969; Crapo et al., 1992). Mice with knocked out SOD1 show lacrimal glands that are smaller, inflamed and aging faster. These are associated with deposits of oxidized material in the LG, reduced tear production, secretory vesicles jam in the acinar cells, and keratitis. All manifestations are accentuated with aging (Kojima et al., 2012).

Throughout recent years, other enzymes and hormones have been identified as prompting antioxidant effects, although their mechanisms are not completely clear. Among them are serine protease inhibitor A3K (SERPINA3K) and erythropoietin (EPO), which is suspected to be produced by the LG and has been proven to be protective against corneal epithelial ROS by activating antioxidant enzymes (Gerina 1980; Zhou et al., 2012; Rocha et al., 2013; Lin et al., 2014).

Peroxiredoxin (PRDX) is a family of six antioxidant enzymes expressed in eye tissues, including the ocular surface epithelial basal layers (the cornea, conjunctiva and the limbus). In comparison to healthy subjects (PRDX1, 2, and 5), it has been more frequently expressed in inflammatory conditions of the ocular surface and in the tear film associated with DE, as shown by pterygium biopsies (peroxiredoxin 1 and 2) (Acera et al., 2011; Soria et al., 2013; Jee et al., 2014; Klebe et al., 2014).

Recently, 481 proteins were identified in human tear film. Among them, 18 are agents that protect against OS, including GPX3, and 7 are related to immune defense (Table 1) (de Souza et al., 2006).

**TABLE 1:** Genetic source and comparison of antioxidants and free radical scavengers identified in the lacrimal gland and ocular surface cells or tear secretion of human, mouse, rabbit or rat (Flicek 2020).

Peroxidase	Species	Chromosome	Gene	Number of transcripts	Size (bp)	Protein length (aa)
GPX1	Human	3	49,357,176–49,358,358	8	1,135	98
	Mouse	9	108,338,903–108,340,343	4	1,072	201
	Rat	8	117,117,430–117,118,522	1	873	200
	Rabbit	9	16,889,533–16,890,546	1	765	200
GPX3	Human	5	151,020,438–151,028,992	11	1,603	226
	Mouse	11	54,902,453–54,910,377	3	1,594	226
	Rat	10	40,247,436–40,255,422	1	1,468	225
	Rabbit	3	32,604,587–32,612,826	3	1,899	209
LPO	Human	17	58,218,548–58,268,518	11	2,821	712
	Mouse	11	87,806,428–87,828,289	3	2,926	710
	Rat	10	75,100,385–75,120,247	1	2,930	698
	Rabbit	19	29,990,709–30,006,671	1	2,133	710
Myeloperoxidase (MPO)	Human	17	58,269,855–58,280,935	5	3,216	745
	Mouse	11	87,793,581–87,804,413	6	2,750	718
	Rat	10	75,087,892–75,098,260	3	2,447	718
	Rabbit	19	29,979,881–29,987,807	2	2,178	725
Prostaglandin H Synthase (PTGS1 or PGHS)	Human	9	122,370,530–122,395,703	10	5,020	599
	Mouse	2	36,230,426–36,252,272	5	2,867	602
	Rat	3	15,560,712–15,582,344	1	2,803	602
	Rabbit	GL018699	4,844,439–4,872,392	7	2,450	618
Thyroid Peroxidase (TPO)	Human	2	1,374,066–1,543,711	16	4,085	933
	Mouse	12	30,054,659–30,132,624	3	3,299	914
	Rat	6	49,021,044–49,089,855	1	3,238	914
	Rabbit	GL019082	1,287–63,437	6	3,583	642
Peroxiredoxin 1 (PRDX1)	Human	1	45,511,036–45,523,047	6	1,234	199
	Mouse	4	116,685,544–116,700,822	6	2,294	199
	Rat	5	135,536,413–135,551,990	1	889	199
	Rabbit	13	121,125,072–121,135,656	2	830	199
Peroxiredoxin 2 (PRDX2)	Human	19	12,796,820–12,801,910	6	925	198
	Mouse	8	84,969,587–84,974,834	8	1,612	198
	Rat	19	26,084,903–26,090,094	1	876	198
	Rabbit	no orthologs				
Peroxiredoxin 5 (PRDX5)	Human	11	64,318,121–64,321,811	3	860	214
	Mouse	19	6,906,697–6,910,106	5	1,262	210
	Rat	1	222,164,462–222,167,447	1	816	213
	Rabbit	no orthologs				

Reference: http://www.ensembl.org/

All these findings lead to two paradoxes: 1) OS is present in the ocular surface simultaneously for protection and harm; 2) increase in the OS biomarker indicates health and healing capacity or tissue degradation and the lack of organ homeostasis.

To deeply understand these paradoxes, it is important to evaluate the biomedical literature in the following topics: 1) available peroxidase assays and limitations; 2) clinical evidence of a relationship between ROS and disease; and 3) potential therapeutic approaches.

## Limitations of Each Antioxidant or Oxidative Stress Assay in the Lacrimal Gland, Ocular Surface, and Tear Film

Under physiological conditions, oxidant-antioxidant balance or redox homeostasis is equalized with minimum rest (Droge 2002). Signs of waste can be observed when comparing biologic markers of oxidative stress in aged versus young organisms, as well as on the ocular surface (Batista et al., 2012; Cejka and Cejkova 2015; Forman 2016).

Limitations in tracking the events associated with healthy and pathologic OS using biologic and chemical *in vitro* and *in vivo* assays are rarely mentioned. Approximately 0.1% of the O_2_ reduced during normal cell metabolism generates free radicals. Meanwhile, 1–3% of all electrons in the transport chain “leak” to generate superoxide and contribute to pathologic events instead of contributing to the transformation of oxygen into water (Imlay and Fridovich 1991; Valko et al., 2007).

The limitations begin in the fact that those molecules and their free radical metabolites, including superoxide (O^−^), nitric oxide (NO), hydrogen peroxide (H_2_O_2_), and hydroxyl (OH^−^), are labile. Therefore, measurement in live tissues, although feasible, is not easy or free from artifacts (McCord 1974; Looms et al., 2002; Hodges et al., 2005).

Currently, the main source of OS and the presence of antioxidants or free radical scavenger enzymes are tested or confirmed in tissues and fluids in different ways, with different specificities (Table 2).

**TABLE 2:** Assays and their limitations for OS, antioxidant or free radical scavenger expression or activity in the LG, tear film or the ocular surface.

Reference	Goal	Method	Detected Product	Specificity	Limitations
Morrison and Allen (1966)	Identify LPO in LG	Rabbit immune serum against LPO and electrophoresis	Immunoprecipitation in electrophoretic gel	Low	No quantification or cell localization
(Herzog and Miller 1972b)	Determine the location of endogenous peroxidase in acinar cells and fluid	Biochemistry and electron microscopy	Dark spots on electron microscopy and colorimetric assay with guaiacol and pyrogallol test	Low	Limited distinction among peroxidases and catalase activity
Herzog and Fahimi (1976)	Distinguish peroxidase and catalase in acinar cells	Optimum pH evaluation, glutaraldehyde activity to selectively inhibit catalase or peroxidase and electron microscopy	Peroxidase activity and dark spots in electron microscopy	Moderate	Limited distinction from other peroxidase enzymes
Herzog et al. (1976)	Measure the peroxidase release under pharmacologic modulation from the LG cells	Histological and biochemical response to α-adrenergic and muscarinic cholinergic stimulus	Location of dark spots in the acinar cells *in vitro* and measurement thereof in the tissue and in the supernatant by DAB colorimetric assay	Moderate	Limited distinction among peroxidases isoforms
De (1992)	Measure tissue peroxidase activity and confirm tissue location	Peroxidase purification from tissues and rabbit antiserum anti peroxidase production for western blotting	Comparative enzymatic tissue assays and tissue presence confirmation	Moderate	Potential cross-reaction on western blotting analysis and enzymatic assays
Rios et al. (2005)	Compare LG peroxidase activity as biomarker of DE	Colorimetric assay to obtain peroxidase activity in LG tissues	Colorimetric assay of peroxidase activity	Moderate	Limited distinction among peroxidases isoforms
Jee et al. (2014)	Measure antioxidant enzymes in the tear film	Luminex: magnetic bead-based immunoassay	Catalase and SOD in tear secretion	High	Amount of tear sample and external contamination
Sakai et al. (2015)	Observe the conjunctiva cell culture in the absence of specific antioxidant enzymes	Knockdown of antioxidant enzymes with small interfering RNA	Lipid oxidation and ROS measured by LDH method	High	Immortalized cells in culture have limited compensatory mechanisms
Soria et al. (2017)	Comparative expression of peroxidase and other tear film contents in different diseases	Liquid chromatography with tandem mass spectrometry and quantitative analysis with spectral APEX proteomics	Identification and quantification of enzymatic Redox markers in the tears	High	Tear volume availability and collection method

DAB: 3-3 diaminobenzidine tetrahydrochloride; LDH: lactate dehydrogenase.

Considering their association with many diseases, a large number of studies have investigated the presence and activity of antioxidant enzymes, the presence of their mRNA and deposits or their residual metabolites of OS reactions as indirect biomarkers of diseases (Marcozzi et al., 2003; Atli et al., 2004; Nogueira et al., 2005; Adachi et al., 2006; Kurimoto et al., 2007; Jorge et al., 2009; Deconte et al., 2011; Knas et al., 2016; Zhang et al., 2017; Seara et al., 2018).

The major concerns are related to the generalization of damage/activity based on biomarker detection and to the nomenclature of the enzymes. “Peroxidase” and “peroxide activity” are terms that have been used disregarding their isoforms/families based on genetic and structural types, specific roles, radical substrates, and cell type expression (Table 2). MPO, LPO and salivary peroxidase belong to the heme peroxidase family. GPO, also expressed in the LG, is part of a different group in terms of both structure and function (Davies et al., 2008; Zamocky et al., 2008; Andrade et al., 2014). Their potential roles in pathologic processes and cross-reactivity in enzymatic assays have not been consistently addressed in current studies (Mansson-Rahemtulla et al., 1988; Thomas et al., 1994; Modulo et al., 2009; Rahimy et al., 2010). A better understanding of their specific roles in healthy conditions and in diseases may help to clarify the paradox of the protective and harmful actions of OS and peroxidases.

Several studies have observed ROS and free radical scavengers limited to the LG, ocular surface tissues and/or tear film. Others did so in association with systemic or environmental diseases as part of the homeostatic, defensive, or pathological mechanisms. We summarized the types of tests in studies involving the LG, ocular surface tissues, and tear film and compared the effects of OS, the amount of metabolites generated, and antioxidant enzyme expression and activity (Table 2). The correlation between the amount of the antioxidant expression at the level of mRNA, protein, their activity *in vitro*, or target tissues, and the OS reaction assumed to take part in the pathological models of diseases that are commonly thought to exist. However, they are rarely demonstrated.

Table 2 presents correlations whenever adequately tested, which would help to clarify the second paradox related to the OS response capacity based on the stocks of the specific enzyme in the tested condition/model.

## Biological Evidence of Reactive Oxygen Species in the Lacrimal Gland and Ocular Surface in Health and Disease Conditions

The LFU includes the LG, ocular surface components and tear film, and it is constantly exposed to triggers of OS. The list includes but is not limited to light, glucose and nitrogen metabolites, high oxygen pressure, pathogens and toxic particles carried in the air or in ocular medications (Brignole-Baudouin et al., 2011b; Torricelli et al., 2011; Alves et al., 2014; Cejkova and Cejka 2015; Lee et al., 2016).

As described above, tissues contain several antioxidants and free radical scavengers, which may control the levels of ROS formation and prevent OS from occurring. Better studied models are those in which LPO presents antimicrobial activity in the tear film as a constitutive enzyme. Additionally, those describing the association between the production of ROS and antioxidant enzyme impairment, diseases associated with DED (such as Sjögren’s syndrome), aging, and hormone and metabolic disturbances such as diabetes mellitus (DM) and hypothyroidism.

LPO antimicrobial activity involves disturbing the stability of bacterial wall proteins, inhibiting microbial respiratory enzymes and interrupting the DNA and RNA synthesis processes of pathogenic microorganisms (Zamocky et al., 2008). These events are more effective against Gram-negative and anaerobic bacteria but also have antifungal and antiviral activities (Carlsson et al., 1983; Yamamoto et al., 1991; Chase and Klebanoff 1992; Lenander-Lumikari 1992; Soukka et al., 1992; Ihalin et al., 2006). LPO transforms ROS into metabolites capable of combating pathogenic agents that interfere with signaling mechanisms related to growth and apoptosis through the PI3K and MAPK pathways (Everse et al., 1991; Daiyasu and Toh 2000; Groeger et al., 2009).

These events are intimately related to LG function and ocular surface protection. However, as a constitutive enzyme of the tear film and potential marker of healthy tear secretion and function, there is a lack of understanding of the role of peroxidases in antimicrobial function versus antioxidant metabolic activities in the LFU. This is due to limitations on bioassays conducted in several studies in the past (listed in Table 2), involving cross-reaction and misleading interpretations, as observed before with other biological fluids (Bonini et al., 2007).

The LPO levels in tears decrease with aging in mice and rats (Bromberg and Welch 1985; Bromberg et al., 1989; Rios et al., 2005). Although it is undisputable that aging deteriorates the LFU, antioxidant and innate defenses while ROS accumulates in the tissues, the assays did not specifically investigate enzyme activity for either purpose.

The LG shows higher peroxidase activity in scavenging H_2_O_2_ than pancreatic islets of Langerhans in rats. This suggests that the LG has a better system to respond to oxidative stress and therefore better resistance to toxic and inflammatory agents such as streptozotocin (Cunha et al., 2007).

Regarding the LG in a diabetic rat model, our previous studies revealed that peroxidase activity is higher in the LG tissue of rats with DM induced by streptozotocin than in the LG tissue of the control rats and the insulin- or aspirin-treated DM rats matched for age and sex (Jorge et al., 2009; Modulo et al., 2009). These findings suggest that GPO, a housekeeping enzyme, but not LPO, a secretory enzyme, is responsive to hyperglycemic oxidative damage. However, the presence of higher amounts of lipid peroxidation markers, such as lipofuscin and malondialdehyde (MDA), which are late-phase markers of lipid peroxidation, reveals that higher peroxidase activity is not sufficient to prevent OS.

Not only type 1 DM but also insulin resistance induced by a high-fat diet in rats increased the levels of ROS in salivary glands, parallel to a reduced salivary flow rate. This finding indicated that the parotid gland is the most vulnerable to OS (Knas et al., 2016; Kolodziej et al., 2017).

Moreover, the peroxidase release from the LG of diabetic rats (measured by a method intended to identify LPO) indicated a lower level of LPO in the LG and in the basal region and stimulated tear secretion from those who removed the LG and had fewer secretory granules *ex vivo* (Shetty et al., 2009). Those studies showed discrepant results on peroxidase activity in the same animal model of DM, where higher OS and higher peroxidase activity are not associated with higher LPO secretion. These findings reveal the need to distinguish the metabolic response to ROS and the effect on secretory activity concerning different peroxidase enzymes and activity in the LG. In DM, the former (high OS) was likely observed in events predominantly associated with GPx versus ROS, and the latter (weaker LG secretory activity) was observed in those events associated with impaired secretory activity related to LPO.

We compared the expression of LPO, GPx and LF proteins by western blotting in the LG of healthy and diabetic rats (after 8 weeks of streptozotocin induction). No significant differences were detected in their expression, which suggests that the expression of those enzymes was not severely affected by DM. Alternatively, they were cross-reacting and hindering the identification of changes in each of those enzymes (data not shown) (De 1992). A better distinction among various antioxidant enzyme expression and activity will be useful to clarify those discrepancies.

A higher activity of GPx in the parotid gland compared to the submandibular gland of individuals with DM was also associated with higher levels of OS (Nogueira et al., 2005; Kolodziej et al., 2017). Recently, additional evidence of the association between DM and DE involving OS was described in high-fat diet-induced DM in mice, where reductions in tear flow and corneal epithelial thickness were observed. In parallel, after 8 weeks, there was an initial increase followed by subsequent declining levels of the following antioxidants in the corneas of diabetic mice with DE: silent information regulator 1 (SIRT1), fork head Box O 3 (FOXO 3), and Mn superoxide dismutase (MnSOD). This suggests that these molecules act in a particular pathway in response to OS related to DE in the cornea (Liu et al., 2015). In human corneal epithelial culture cells and rat corneal cells *in vivo* challenged with H_2_O_2_, the higher expression of catalase and SOD induced by SERPINA3K minimized the OS damage to those epithelial cells (Zhou et al., 2012).

In another DE model induced by hormone impairment (hypothyroidism induced by thiamazole in rats), lipid peroxidation was detected in ocular surface tissues and tear film (Drozhzhyna G.I. & G.I. 2019). This finding agreed with previous work in which male rats with hypothyroidism presented lower tear secretion, lower peroxidase activity, and increased levels of malondialdehyde in the LG (Dias et al., 2007). Both studies also confirm the associated repercussions in the tissues integrated by the LFU.

Considering the sex differences in the LFU in health and disease, the possible modulation of ROS by sex hormones and their implications on DE, it is interesting to observe that the LG of a female hamster has three times more peroxidase activity than the male LG. Further studies revealed that it could be suppressed by androgen (Paliwal and De 2006; Dowling and Simmons 2009; Sullivan et al., 2017). To confirm these findings in rats, we compared the activity of peroxidase between the LG homogenates in the male and female rat by a colorimetric test, but no difference was found (Table 3). To investigate whether peroxidase enzyme levels would change and could overlap with the activity of the other, we used western blotting and specific antibodies (Santa Cruz Biotechnologies, Santa Cruz, CA, United States) to compare the expression of three enzymes involved in OS in the LFU, GPx3, LPO, and LF. All of them presented similar levels in the LG of 8-week old female and male Wistar rats (data not shown). Thus, these results limit our understanding of the role of sex hormones in oxidative stress enzymes in DE and LFU diseases.

**TABLE 3:** Peroxidase activity in Wistar rats LG. The comparison included eight-week-old males and females (*n* = 7/group). The assay was conducted with a colorimetric assay (*Amplex Red*; Molecular Probes, Eugene, OR, United States) and read at a spectrophotometer (SpectroMax M2, Molecular Devices, Sunnyvale, CA, United States) after 30 min. Comparisons were performed by Student’s t test for the two groups and ANOVA for the three groups. The *p* value was significant when <0.05.

	Peroxidase activity mU/ml	*p* value for male versus female	Peroxidase activity/LG weight mU/ml.mg	*p* value for male versus female
Male	395.6 ± 90.5	0.88	4.3 ± 0.9	0.96
Female	380.6 ± 71.5		4.3 ± 0.8	

Nonobese diabetic (NOD) mice have been used in studies to understand DM and Sjögren’s syndrome-related DE (SSDE). Their characteristics reveal that females are more likely to develop DM and males are more likely to develop LG but not salivary gland (SG) inflammation (Toda et al., 1999; de Paiva and Rocha 2015). The possible role of antioxidant enzymes was not explored in the LG of NOD mice. However, the expression of antioxidant enzymes (including SOD, catalase and GPx) in various tissues, such as lung and pancreatic beta cells, is higher in males. This suggests that they have better protection against pancreatic destruction (Cornelius et al., 1993). Moreover, transgenic overexpression of thioredoxin antioxidant enzyme in pancreatic beta cells prevented the onset of DM in NOD mice (Yamamoto et al., 2008). Whether sex differences in SSDE NOD mice have any relationship with OS is still unknown.

The commensal microbiota has a protective and anti-inflammatory effect on the ocular surface and the LG compared to germ-free conditions in wild-type C57BL/6J and CD25 knockout mice, which is a model for SSDE (Wang et al., 2018; Zaheer et al., 2018). The mechanisms of action of the gut microflora in such events are not clearly known. Only five key communication routes are described as hypotheses that relate ocular surface diseases and gut dysbiosis: myeloid cell migration (altered migration of dendritic cells or macrophages to the lymph nodes, ocular surface and the lacrimal gland to prime T cells or promote pro-inflammatory cytokines secretion); effector lymphocyte imprint (T_reg_/T_H_17 imbalance in ocular surface and LG); molecular mimicry (B cells autoantibodies production mediated by microbial-derived antigens); metabolite circulation (the reduction of short-chain fatty acids microbial metabolites, which affect the tear secretion) and neuropeptide circulation (gut-derived neuropeptides influence tear secretion). However, as mentioned above, mechanical investigations are necessary to further elucidate these five key communication routes and their potential overlap (Thaiss et al., 2016; Moon et al., 2020). The effect of the microbiota inducing a balanced level of OS to modulate the innate immune system and promote homeostasis in the other organs and systems is considered part of the protection mechanism. Indeed, dysbiosis is a deviation in the communication of the gut-brain axis that induces exaggerated inflammation and OS (Luca et al., 2019).

The best and most long-standing agreement among studies regarding the LFU and OS is seen in aging models. The LG of old mice and rats present lower peroxidase activity and lower stimulated peroxidase secretion than their younger counterparts (8 weeks old) (Bromberg et al., 1989; Alves et al., 2005b; Rios et al., 2005). These observations are also in agreement with functional impairment of secretion, reduction of molecules associated with secretory control such as Rab3d, accumulation of degenerative and oxidative stress markers such as lipofuscin, and reduction of vitamin E (an antioxidant) in the LG of aged rats (Batista et al., 2012). Functional changes were prevented in aging rats with 35% calorie restriction (CR), which also preserved the tissue structure, reduced inflammatory cell infiltration, and attenuated the markers of oxidative damage to the LG (Kawashima et al., 2010). In autoimmune mouse models, 40% CR attenuated the inflammatory process in the SG, extended their lifespan, and, in healthy primates, delayed degenerative diseases (Chandrasekar et al., 1995; Colman et al., 2009). The mechanism is attributed to OS reduction in metabolic and inflammatory processes (Heilbronn and Ravussin 2003). In SOD knockout mice, functional impairment and increases in several degenerative parameters are associated with aging. This included OS and inflammatory markers in the LG and tear film, manifested as early as 50 weeks of age, indicating that antioxidant enzymes and OS have a key role in the causative events related to age-related DE (Kojima et al., 2012). Observations that ROS trigger DE pathology were also identified in a *mev-1* conditional transgenic mouse (*Tet-mev-1*), in which mitochondrial oxidative damage led to DE through LG damage (Uchino et al., 2012). Those last observations indicating OS as a causative factor are relevant to the understanding of DE physiopathology, avoiding the chicken and egg dilemma or the belief that ROS are only a side effect of other causes of DE. These findings could support the goal of therapeutic strategies focused on antioxidant and OS prevention.

Meanwhile, OS can be considered an adjuvant factor in the mechanism of DE and ocular surface diseases. Although studies identified the suppression of antioxidant activities as triggers of DE, the action of genetic knockout enzymes may produce other nontracked effects associated with it. Moreover, all other models, including DM, hormone deficiency, and aging, present several competing factors, such as impaired neural transmission and metabolism, hypotrophism, and osmotic imbalance (Rocha et al., 2003; Alves et al., 2005a; Alves et al., 2005b; Kojima et al., 2012; Uchino et al., 2012; Dias et al., 2015). Therefore, it is unlikely that OS induced by a deficiency in antioxidant enzymes in the LFU is the only cause of DE in major conditions such as aging, DM, and hormone deficiency. It is more plausible that OS is an adjuvant factor.

## Clinical Evidence of Reactive Oxygen Species as a Cause of Dry Eye

Sex differences in LG and DE diseases, with a higher prevalence in females, are remarkable characteristics that are well reviewed (Sullivan et al., 2017). The investigation of OS in the female sex revealed that peroxidase activity in the tear film is enhanced in response to serum levels of estrogen, and it is also attenuated by oral contraceptives (Liberati et al., 2002). Moreover, peroxidase activity in tears is similar between young men and women and diminishes at a later age in men compared to women, being therefore higher in middle-aged men compared to menopausal women (Marcozzi et al., 2003). Of equal importance, several environmental and behavioral factors may influence sexual comparative analyses of the antioxidant capacity of tears, namely, smoking habits and exercise frequency (Gus et al., 2006).

**FIGURE 2:**
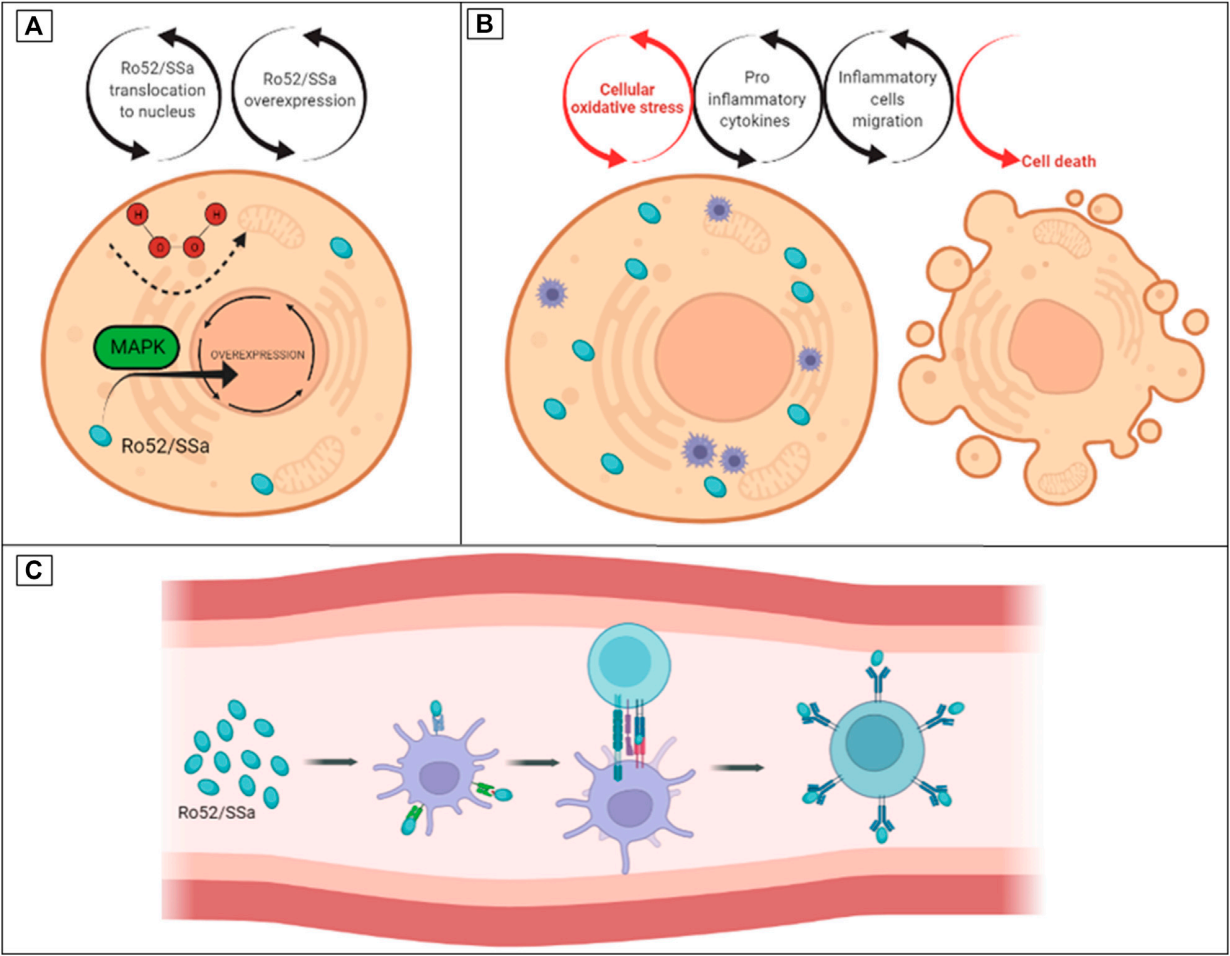
The hypothesis of turning Ro52/SSa from an oxidative stress intracellular signaling molecule to an auto antigen in SS. **(A)** Ro52/SSa translocation to the nucleus by H_2_O_2_ stimuli. **(B)** Overexpression of Ro52/SSa led to cytotoxicity and inflammation. **(C)** Immune sensitization and production of anti-Ro autoantibodies.

Sjögren’s Syndrome (SS) is a systemic autoimmune disease more prevalent in women, with an unknown cause and no cure, that causes DE and dry mouth due to the inflammation of exocrine glands (Akpek et al., 2011). Major biomarkers of SS are autoantibodies that protect against intracellular ribosomal proteins, including anti-Ro52/60, also called SSa (Vitali et al., 2002). It was demonstrated that Ro52/SSa is a signaling molecule sensitive to H_2_O_2_ that is capable of translocating from the cytoplasm to the nucleus in the MAP kinase (MAPK) pathway (Nobuhara et al., 2007). Other studies associate the autoantibodies of SS (SSa and SSb) with cross-reactivity of viral proteins. There is plausibility for the hypothesis that excessive OS and overexpression of signaling molecules such as MAPK lead to cytotoxicity and inflammation. Subsequent cytolysis induces exposure and immune sensitization to intracellular molecules and the production of autoantibodies as part of the complex mechanism in the target tissues of SS disease (e.g., exocrine glands) (Figure 2) (Baboonian et al., 1989; Scofield and Harley 1991; Garcia-Carrasco et al., 2006).

Markers of OS, such as 8-hydroxy-2′-deoxyguanosine (8-OHdG) and hex-anoyl-lysine (HEL), present higher levels in the saliva of SS individuals than in other salivary dysfunctions (Ryo et al., 2006). These observations were later confirmed and accompanied by higher levels of the antioxidant thioredoxin in the saliva of SS individuals compared to the control group (Kurimoto et al., 2007). In systemic sclerosis, another autoimmune disease commonly associated with SS, impaired peroxidase activity and reduced salivary levels of SOD are related to reduced unstimulated salivary flow. In individuals with normal unstimulated salivary flow, the values were similar to those of the control group (Zalewska et al., 2014). In rheumatoid arthritis (RA), blood levels of antioxidant enzymes and OS markers vary among studies (Quinonez-Flores et al., 2016). Together, these observations indicate the association of OS and inflammatory processes in autoimmune diseases. Discrepancies among studies may reflect differences along the disease time-course.

Tear proteomics was capable of identifying eighteen antioxidant enzymes in the tears of healthy volunteers (de Souza et al., 2006). Impression cytology analysis revealed that the conjunctival epithelial cells in patients with SSDE express lower amounts of SOD, GPx, and catalase than in healthy controls (Cejkova et al., 2008). However, tears of SS individuals have higher average levels of proteins than non-SS DE and controls. Tear proteomics also revealed that proteins related to OS (including SOD) and inflammatory activities presented higher levels in SS (Li et al., 2014). Differences in antioxidant enzyme expression, such as SOD, in SS individuals in comparison to controls may reflect aspects of the examined tissue, the time course of SS disease, and the methods utilized. Nonetheless, these differences indicate the fragility of using those biomarkers to understand complex aspects of OS in the LFU.

Lipid peroxidation markers such as HEL and 4-hydroxy-2-nonenal (4-HNE) were identified in the conjunctiva. The former was also identified in the tears of SS individuals. It was positively correlated with poor scores in clinical ocular surface findings and with the number of inflammatory cells harvested by brush cytology (Wakamatsu et al., 2013). These observations were later confirmed in a large sample of non-SS individuals who were compared to controls using other lipid peroxidation markers, suggesting that they are not present solely in SS (Choi et al., 2016b).

The mentioned findings indicate that OS markers are present in the saliva and tears of SS individuals and may take part in the mechanism of the disease. At the time when SOD and catalase were identified in joints and shown to protect synovial fluid degradation by ROS during inflammatory processes, therapeutic approaches against OS were suggested (McCord 1974). The actual stage of protection provided by antioxidants will be discussed in the next section.

## Potential Therapeutic Approaches and the Role of Nutrition

There are controversies regarding biomarker identification and the role of OS as a trigger and not only a side effect or associated event of tissue damage and LFU dysfunction in patients with DE. However, many studies have emphasized the preventive and therapeutic approaches of antioxidants for patients with DE (Table 4 and Table 5).

**TABLE 4:** Preventive and therapeutic antioxidant strategies for DE disease in animal models published between 2010 and 2019 were searched for “antioxidant” and “dry eye” in PubMed.

Animal model	Study design; number of individuals	Treatment	Parameter studied	Benefit (yes/no)	Reference
Rabbit	Experimental research	Poly (catechin) capped-gold nanoparticles carrying amfenac; ED	Ocular surface tissue damage	Yes	Li et al. (2019)
*In vivo*: mice	Case control study and experimental research	Manganese (III) tetrakis (1-methyl-4-pyridyl) porphyrin,ED	CFS, leukocyte infiltration into the LG and parenchymal tissue degeneration	Yes	Ziniauskaite et al. (2019)
*In vitro:* HCEC					
6-week-old male Sprague–Dawley rats	Case control study (*n* = 10/group)	Seleno protein P, ED, 5 or 50 mg/ml, 6 times per day for 3 weeks	CFS	Yes	Higuchi et al. (2010)
*In vivo:* six-week-old male Wistar rats	Case control study and experimental research	Polydatin, ED 0.05% or 0.5%, 3 times a day	TV, TFBUT, corneal irregularity, LGS, CFS, histology, immunohistochemistry	Yes	Park et al. (2019)
*In vitro:* HCC					
*In vivo:* seven-week-old male Wistar rats	Case control study and experimental research	*Polygonumcuspidatum,* orally administered 10, 100 or 250 mg/kg	TV, cornea irregularity, immunohistochemistry cell viability	Yes	Park et al. (2018)
*In vitro:* HCEC					
Six to eight-week-old C57BL/6 female mice	Case control study (*n* = 10/group)	0.001, 0.01, or 0.1% plant extracts ED, 2 μL, 3 times a day, for 10 days	TV, TFBUT, CFS	Yes	Choi et al. (2016a)
Six-week-old male Sprague–Dawley rats	Experimental research	0.01, 0.1, or 1% Se-lactoferrin ED contained 1.8, 18, or 180 µM selenium, respectively, 4 times per day for 2 weeks	CFS	Yes	Higuchi et al. (2012)
Six-week-old male Sprague–Dawley rats and albino rabbits	Case control study (*n* = 12/group) and experimental research (rabbits)	0.1% Se-lactoferrin ED containing 18 μM selenium, 4 times per day for 5 days in mice	CFS	Yes	Higuchi et al. (2016a)
Six-week-old male Sprague–Dawley rats	Case control study (*n* = 10/group)	0.1, 1 and 10 µM of 2-hydroxy-estradiol	CFS, TV	Yes	Higuchi et al. (2016b)
Six to eight-week-old female C57BL/6 mice	Case control study (*n* = 5/group	0.1% hyaluronic acid alone or mixed with 0.1, 0.5, or 5.0% mineral oil ED	TV, corneal irregularity score, TFBUT, and CFS	Yes	Choi et al. (2015)
Six-month-old male Fischer 344 rats	Case control study	Calorie restriction, 6 months	TV, histological examination, tear protein secretion stimulation test with Carbachol, and assessment of 8-OHdG and HNE antibodies	Yes	Kawashima et al. (2010) ^24^
6-month-old pigmented rabbits	Case control study	Mitochondria-targeted antioxidant, 7.5 μM topical administration	CFS, histological analysis, ST, and TFBUT	Yes	Zernii et al. (2017)

HCEC: human corneal epithelial cells; ED: eye drops; CFS: corneal fluorescein staining; HCC: human conjunctival cell; TFBUT: tear film breakup time; TV: tear volume; LGS: lissamine green staining; 8-OHdG: oxidative stress with 8-hydroxy-2, deoxyguanosine; HNE: 4-hydroxynonenal; ST: Schirmer’s test.

**TABLE 5:** Preventive and therapeutic antioxidant strategies for DE disease in humans published between 2010 and 2019 were searched for “antioxidant” and “dry eye” in PubMed.

Human (disease when specified)	Study design; number of individuals	Treatment	Parameter studied	Benefit (yes/no)	Reference
Severe kerato conjunctivitis sicca	CCS; 43 individuals	Artelac Rebalance (cyanocobalamin), ED, 12 months	ST, TFBUT, LO, CFS and CS, meniscometry, CCT, and OSDI	Yes	Safonova et al. (2019)
DE disease	CCS; 118 individuals	Hydrogen-producing milk, once/day for 3 weeks	TFBUT, ST, 8-OHdG concentration in tears, DES, and VA	Yes	Kawashima et al. (2019)
DE disease and corneal wounds	Experimental research; conjunctival epithelial cells	Visomitin, 300 nM (181 ng/ml) for 24 h	Production of inflammatory biomarker prostaglandin E2 and cell viability	Yes	Wei et al. (2019)
DE disease	Experimental research; corneal epithelial cells	Epigallocatechin gallate component of green tea at 0,3–30 µM	Cell metabolic activity, MAPKP, glucose oxidase-induced ROS production	Yes	Cavet et al. (2011)
Moderate-to-severe DE disease	CT; 349 individuals TG and 186 in PG	Omega-3 fatty acid, 3,000 mg daily oral dose, for 12 months	OSDI, CS and CFS, TFBUT, and ST	No	Dry Eye et al. (2018)
DE disease	CCS; 20 individuals in TG and 23 in PG	Antioxidant supplement oral, for 8 weeks	DES, VA, ST, TFBUT, CS and CFS, serum anti-SSA/anti-SSB antibodies, and the level of ROS in tears	Yes	Huang et al. (2016)
DE disease	CCS; 16 patients with severe DE and 17 healthy controls	Samples of 50% autologous serum ED	Total reactive antioxidant potential and concentration of ROS	Yes	Gus et al. (2016)
DE disease	CCS; patients with DE no treatment (*n* = 29) or treatment with hyaluronic acid/vitamin B12 ED (*n* = 32), and patients without DE (*n* = 42)	Preservative-free ED containing hyaluronic acid 0.15% and vitamin B12	ST, fluorescein clearance test, TFBUT, and OSDI	Yes	Macri et al. (2015)
DE disease	Observational cross-sectional study; 16.396 participants	Serum 25-hydroxyvitamin D levels	Standardized interviews, blood 25-hydroxyvitamin D level evaluations, and ophthalmic examinations	No	Jee et al. (2015)
Moderate-to-severe DE syndrome	CCS; 50 individuals in each group	Preservative-free ED	DES, TFBUT, ST, CFS, and CIC	Yes	Jee et al. (2014)
Nonsevere DE disease	CCS; 30 individuals with DED and 32 healthy	Nutraceutical formulation based on the combination of antioxidants and ω-3 essential fatty acids, orally	OSDI, VA, ST, TFBUT, and CFS	Yes	Pinazo-Duran et al. (2013)
DE disease	Open-label, prospective, noncomparative, intervention, and multicenter study; 1,419 individuals	Nutraceutical formulation containing omega-3 fatty acids, vitamins, minerals, and antioxidants, orally, 3 capsules/day, 12 weeks	DES, conjunctival hyperemia, TFBUT, ST, and OGS	Yes	Gatell-Tortajada (2016)
DE disease	CCS; 55 individuals diagnosed with DE and 35 healthy subjects	Nutraceutical supplementation containing antioxidants and essential polyunsaturated fatty acids, orally, 3 capsules/day for 3 Months	Metabolomic profiles in tears through nuclear magnetic resonance spectra	Yes	Galbis-Estrada et al. (2015)
Mild DE disease	CCS; 25 individuals in each group	Antioxidant glasses containing extracts of medicinal plants, 15 min, 3 times/day	OSDI, TFBUT, and ST	Yes	Choi et al. (2015)
Moderate-to-severe keratoconjunctivitis sicca in postmenopause	CT; 38 individuals	Gamma-linolenic acid and omega-3 Polyunsaturated fatty acids, orally	OSDI, ST, TFBUT, CFS and LGS, and topographic corneal	Yes	Sheppard et al. (2013)
Moderate DE disease	CCS; 180 individuals	Combination of omega-3 and omega-6), twice a day for 6 months, orally	Dryness feeling, overall subjective comfort, and DES, TFBUT, OGS, and LGS	Yes	Creuzot-Garcher et al. (2011)
Mild-to-moderate DE disease	CCS; 138 individuals	Supplement containing omega-3 andomega-6 fatty acids, vitamins and zinc, 3 capsules/day, for 3 months, orally	Impression cytology to assess the percentage of cells expressing HLA-DR and to evaluate fluorescence intensity, DES, and objective signs	Yes	Brignole-Baudouin et al. (2011a)
DE disease	CT; 264 eyes in TP and 254 eyes in PG	Omega-3 fatty acids one capsule (500 mg) 2 times/day	VA, ST, TFBUT, RBS, CIC, and DES	Yes	Bhargava et al. (2013)

CCS, case–control study; CT, clinical trial; DE, dry eye; ST, Schirmer’s test; TFBUT, tear film breakup time; LO, lacrimal osmolarity; CFS, corneal fluorescein staining; CS, conjunctiva staining; CCT, corneal confocal tomography; OSDI, ocular surface disease index; 8-OHdG, oxidative stress with 8-hydroxy-2, deoxyguanosine; MAPKP, mitogen-activated protein kinase phosphorylation; ROS, reactive oxygen species; TG, treatment group; PG, placebo group; VA, visual acuity; DES, dry eye symptoms; ED, eyedrops; RBS, rose bengal staining; CIC, conjunctival impression cytology; OGS, oxford grading scheme.

Strategies to revert or treat DE with antioxidant therapies are becoming more common since 2010, based on the growing number of publications (Seen and Tong 2018; Higuchi 2019).

Regarding animal models, different treatment approaches have been reported, including many chemical substances. Plant extracts diluted in ethanol at different concentrations and applied topically three times a day for 10 days in mice showed that at 0.1%, it improved scopolamine-induced DE parameters (Choi et al., 2016a). Different strategies of administering selenium by topical eye drops to different DE animal models revealed improved parameters of DE and reduced markers of OS, which was also observed in corneal epithelial cells *in vitro.* Examples of these strategies include LG ablation, exposure to tobacco fume in rats and short-term desiccation in rabbits (Higuchi et al., 2012; Higuchi et al., 2016a). The beneficial results of the three studies are attributed to the associated enhancement of GPx activity.

The presence of antioxidants and ROS in 50% human autologous serum eye drop (ASED) samples was investigated. They were found to be similar among individuals with an ocular surface disease and with healthy controls and were stable in 3 months (Gus et al., 2016). This is an interesting observation, since ASED have been used for tear replacement and corneal wound healing for decades. However, their components and ubiquitous mechanisms of action have been seen for a long time (Fox et al., 1984; Leite et al., 2006). In humans, DE and non-DE individuals were compared after 1 month of vitamin B12/hyaluronic acid eye drop use before cataract surgery (Macri et al., 2015). A conjunctival biopsy during the surgical treatment provided tissue to evaluate OS markers by immunohistochemistry. The parameters were compared among the groups and correlated with DE clinical findings. Although a benefit was demonstrated, the lack of a placebo group, the use of lubricant or a vehicle, and the short period of follow-up all limit the conclusion that an antioxidant was a determinant for the obtained results (Macri et al., 2015).

The efficacy of drugs to reduce OS in DE involves careful examination, since the simple change or suppression of benzalkonium chloride (BAK) preservative can reduce the OS in the ocular surface. The BAK suppression in corticosteroid and cyclosporine A eye drops for 30 days was able to reduce the expression of inflammatory markers, increase the presence of antioxidant enzymes, and improve DE parameters in individuals with severe DE compared to controls using preserved formulas of the same eye drops (Jee et al., 2014). On the ocular surface, BAK is itself degraded into H_2_O_2_, leading to OS, inflammation, and tissue damage (Ingram et al., 2004; Baffa et al., 2008; Marques et al., 2015).

Oral treatment with capsules containing vitamins, omega-3, and other antioxidant elements, such as micronutrients, has been tested in DE individuals. The prescription of those supplements as a complementary DE therapy, despite a very modest increase in some of the parameters tested, is encouraged by the minimal risk of side effects (Galbis-Estrada et al., 2015; Gatell-Tortajada 2016; Huang et al., 2016). However, many of those studies have limited evidence-based benefits because of the lack of a placebo group, the limited follow-up period, and other uncontrolled factors that may have favored supplementary therapy. Additionally, they were not confirmed by a large randomized clinical trial (Rosenberg and Asbell 2010; Galbis-Estrada et al., 2015; Gatell-Tortajada 2016; Huang et al., 2016; Dry Eye et al., 2018).

In the same way, the use of glasses containing a plant extract of a combination of medicinal antioxidants for 8 weeks, 3 times a day (15 min/each), compared to a group who used placebo glasses, presented improvement in comparison to baseline on DE parameters and subjective analyses of the discomfort measured by the Ocular surface disease index (OSDI) score. This also suggests some statistical effect, such as returning to the mean (Choi Won et al., 2015).

Among life habits, the most beneficial to reduce OS and LFU inflammation, although with little robust data in human studies, is calorie restriction (CR) (Tsubota et al., 2012). The simplest explanation is that in reducing glucose and carbohydrate intake and calorie burning, ROS formation as side products of energy generation will be reduced. Subsequently, the cascade of tissue scavengers, inflammation, and cytotoxicity will be diminished, preserving function and postponing the aging process. The appropriate time to introduce this strategy and level of restriction in implementing an effective antioxidant therapeutic strategy is unknown. As mentioned above, most of the studies with animals induce CR of 30% or more and very early in life. However, Dr. Kazuo Tsubota reported his personal experience with benefits observed in his middle age (Tsubota et al., 2010).

Another lifestyle habit with potential beneficial antioxidant effects for DE is physical exercise. Although exercise is largely recommended, excessive exercise and a higher caloric demand are less studied factors that may have a dual effect of optimizing the scavenger mechanisms and inducing OS. As of now, the boundaries are unknown (Kruk et al., 2015; Jones et al., 2017).

The pathogenic microbiota that aggravates the signs and symptoms of chronic diseases is called dysbiosis. It is associated with DE in patients with DM and SS, where a more diverse microbiota reduces the severity of inflammation and OS in the target organs of individuals with those diseases (de Paiva et al., 2016; Luca et al., 2019). These observations reinforce the implications of environmental factors such as the influence of nutritional deficiencies in the development of OS associated with DE. They also reinforce the potential benefit of microbiota manipulation in reducing the impact of those diseases.

**FIGURE 3:**
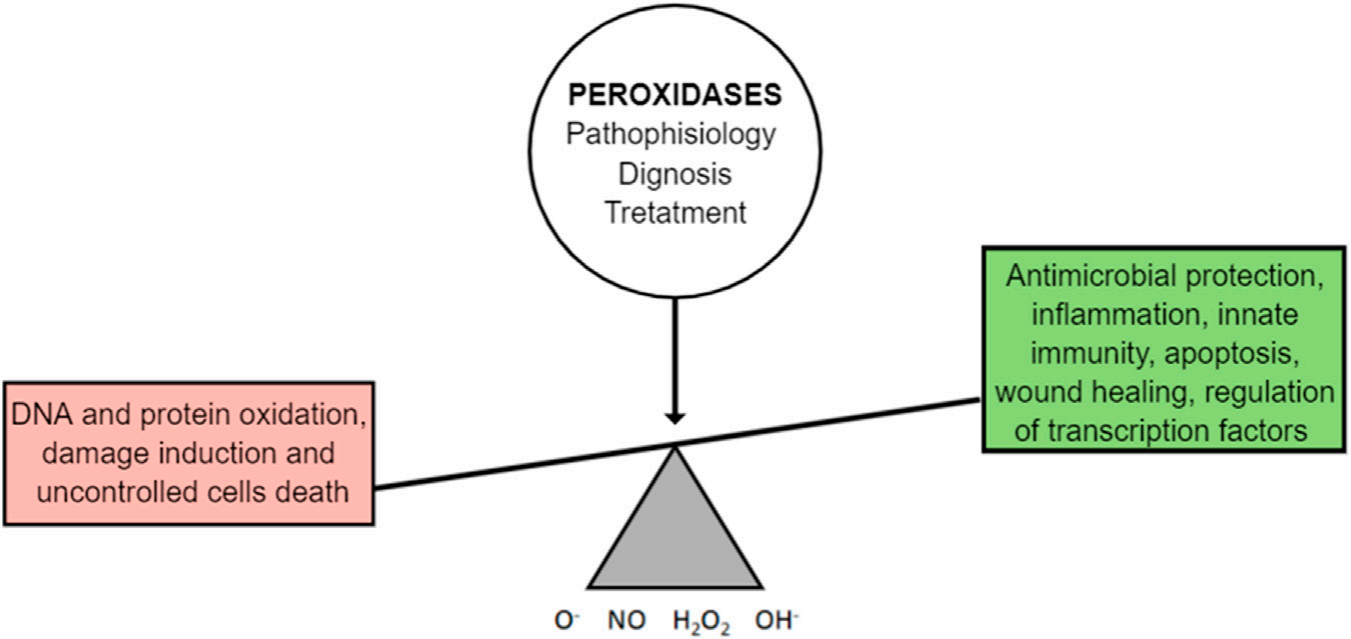
OS is an integrated mechanism of the biological functions and the mechanisms of the diseases. It involves caloric restriction and body synergism with its microflora.

Based on the above, the simplest way to prevent OS related to DE is consuming calorie-restricted healthy meals that are filled with micronutrients and vitamins capable of promoting microbiota diversity, working as ROS scavengers and antioxidants, and limiting the extension of OS in the LFU and in the entire body (Figure 3). However, there are individuals and populations deprived of micronutrients and vitamins in a phenomenon defined as “hidden hunger.” The term is used to differentiate the cases from chronic hunger, where the diets are limited by macronutrients and calories (Gödecke et al., 2018). In fact, the aging population and those with chronic diseases are estimated to comprise approximately two billion people, and the deprivation of such micronutrients and vitamins increases their vulnerability regarding ROS scavenging and antioxidant processes. This may impact DE prevalence and severity, as indicated above (2018).

The causes of hidden hunger involve socioeconomic parameters, cultural habits, modern methods of food production, storage, and processing, and individuals’ health status (Bawa and Anilakumar 2013; Faustino et al., 2016; Faustino et al., 2018; Gödecke et al., 2018; Trono 2019). Genetic modification to produce food crop biofortification enriched for Fe, Zn, and vitamin A is an initiative to revert the global imbalance between an excessive caloric diet versus micronutrient malnutrition. It can be a nutritional approach to the antioxidant strategy of preventing OS in chronic diseases and aging, including DE (Kumar et al., 2019). To date, the impact of dietary micronutrient deficiency on OS associated with DE is unknown. Better biomarkers of OS population studies are necessary to clarify the extent of this association.

In summary, we agree with previous reports that the antioxidant therapeutic approach for DE is not a simple task and that current strategies are limited and require further studies (Seen and Tong 2018).

## Conclusion

In conclusion, it is clear that OS and the expression of the antioxidant elements in the LFU is a growing research field for understanding the mechanisms of DE, the ocular surface response to environmental aggression, and autoimmune mechanisms such as those observed in SS. Biomarkers are still limited in identifying disease mechanisms due to lack of specificity and differences in tissues and species expression, as well as in the duration of diseases. In several models reporting the association of DE and OS, the egg and chicken effect inhibits conclusions about the presence of OS as the cause or as part of the observed impacts, therefore not allowing us to identify exact targets for therapy. Thus far, we do not have among OS markers or antioxidant agents the reliable elements for determining DE diagnoses or exerting DE cures. Therapeutic approaches involve antioxidant therapy and supplementation with micronutrients. The trend of excessive calorie intake associated with micronutrient deficiency may contribute to OS and the associated diseases, supporting nutritional strategies for reducing OS in DE. In the future, the growing understanding of the protective versus the damaging effects of OS in the LFU and the cross-talk between the antioxidant and inflammatory pathways may improve our knowledge of the DE mechanisms and the DE therapy itself.

## Method of Literature Search

A search in the PubMed and Scielo databases was conducted between 2010 and 2020. The search terms of the investigation were “aging,” “antimicrobial,” “antioxidant,” “diabetes mellitus,” “dry eye,” “exocrine gland,” “hormone,” “insulin,” “lacrimal gland,” “lactoperoxidase,” “ocular surface,” “oxidative stress,” “peroxidase,” “saliva,” “sex,” “superoxide dismutase,” “tears” and “tear film.” Our search was extensive, with articles written in English and not limited to the year of publication. All abstracts were screened, and relevant articles were included in this review.
